# Cerebral venous sinus thrombosis concomitant with leptomeningeal carcinomatosis, in a patient with epidermal growth factor receptor-mutated lung cancer

**DOI:** 10.3892/ol.2014.2603

**Published:** 2014-10-10

**Authors:** NAOHIRO ODA, MAKOTO SAKUGAWA, AKIHIRO BESSHO, TAKESHI HORIUCHI, SHINOBU HOSOKAWA, YOSUKE TOYOTA, NOBUAKI FUKAMATSU, KAZUYA NISHII, YOICHI WATANABE

**Affiliations:** Department of Pulmonary Disease, Japanese Red Cross Okayama Hospital, Okayama 700-8607, Japan

**Keywords:** lung cancer, leptomeningeal carcinomatosis, cerebral venous sinus thrombosis, epidermal growth factor receptor-tyrosine kinase inhibitor, magnetic resonance imaging

## Abstract

A 64-year-old woman presented with dizziness, after two weeks of experiencing symptoms. Chest computed tomography revealed a peripheral nodule in her left upper lobe, and brain magnetic resonance imaging (MRI) demonstrated the presence of multiple brain masses. The patient underwent whole-brain radiotherapy based on a tentative diagnosis of lung cancer with multiple brain metastases. The diagnosis was confirmed by endobronchial biopsy as T4N3M1b, stage IV lung adenocarcinoma with an epidermal growth factor receptor mutation. On the 31st day of hospitalization, the patient developed severe headache. Subsequent magnetic resonance venography revealed defects in the superior sagittal, right sigmoid, and right transverse venous sinuses and the right internal jugular vein. Anticoagulation therapy with unfractionated heparin and warfarin was immediately administered following diagnosis of cerebral venous sinus thrombosis (CVST). Brain MRI demonstrated leptomeningeal gadolinium enhancement in front of the pons and medulla. Positive cerebrospinal fluid tumor cytology confirmed the diagnosis of leptomeningeal carcinomatosis. Following four weeks of antithrombotic therapy, complete thrombolysis was confirmed by magnetic resonance venography. Effective treatment with gefitinib was administered, and the patient survived for 10 months after the diagnosis of CVST and leptomeningeal carcinomatosis. Adequate early diagnosis and treatment of CVST enabled an excellent survival rate for the patient, despite leptomeningeal carcinomatosis. Following the development of headaches in patients with lung cancer, CVST, although rare, should be considered. Furthermore, following a diagnosis of CVST, leptomeningeal carcinomatosis should be investigated as an underlying cause.

## Introduction

Cerebrovascular disease is the one of the most frequent cancer complication of the central nervous system, second to brain metastasis (1). The largest study thus far, found that 14.6% of 3,426 cancer patients had cerebrovascular disease based on pathological autopsy results, and ~50% of them were symptomatic (1). Cerebrovascular complications often affect the prognosis of cancer; thus, diagnosis and treatment of these associated pathologies is important. Cerebral venous sinus thrombosis (CVST) is a rare disease of the cerebrovascular system, and there have been few reports of cancer patients with CVST (2). A rare case of leptomeningeal carcinomatosis (LC) is presented, which may be an underlying cause for CVST in patients with lung cancer.

## Case report

A 64-year-old female was referred to the Japanese Red Cross Okayama Hospital (Okayama, Japan) with complaints of experiencing dizziness for two weeks. The patient was not prescribed any medications and had no specific family history. She had smoked one packet of cigarettes a day for 40 years. A neurological examination revealed a mild cognitive impairment (mini-mental state examination, 21/30), left-side fixation nystagmus, and an ataxic gait. Chest computed tomography (CT) revealed an ~3-cm peripheral nodule in the left upper lobe, and brain magnetic resonance imaging (MRI) demonstrated the presence of multiple brain masses in the bilateral cerebrum, cerebellum and pons. Hematological, coagulation, biochemical, and serological findings were normal, except for elevated carcinoembryonic antigen (CEA) and sialylated SSEA-1 antigen levels of 115.8 ng/ml and 32.5 U/ml, respectively. Whole-brain radiotherapy was conducted (30 Gray total) in 10 fractions for two weeks with intravenous dexamethasone and concentrated glycerin, based on a tentative diagnosis of lung cancer with multiple brain metastases.

**Figure 1:**
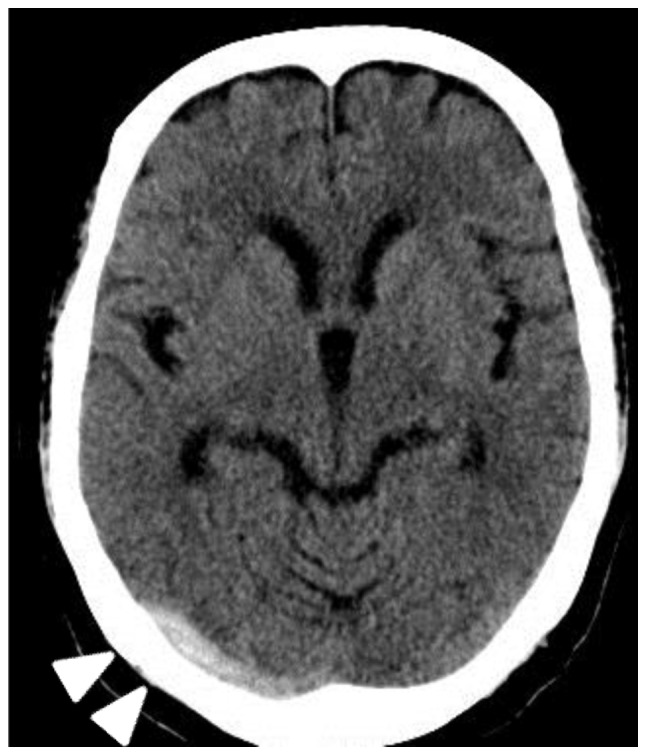
Computed tomography on the 33rd day of hospitalization. A hyperdense area at the right transverse venous sinus is shown (arrowheads).

**Figure 2:**
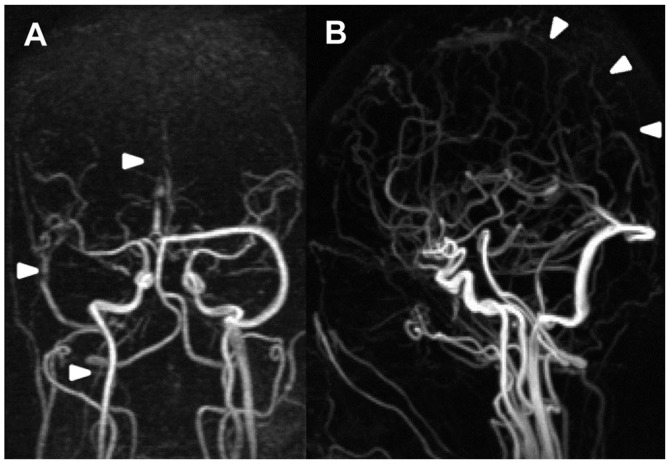
Magnetic resonance venography (MRV) on the 33rd day of hospitalization. (A) Coronal view. (B) Saggital view. MRV confirmed thrombosis (arrowheads) of the sagittal, right sigmoid and right transverse venous sinuses in addition to the right internal jugular vein.

**Figure 3:**
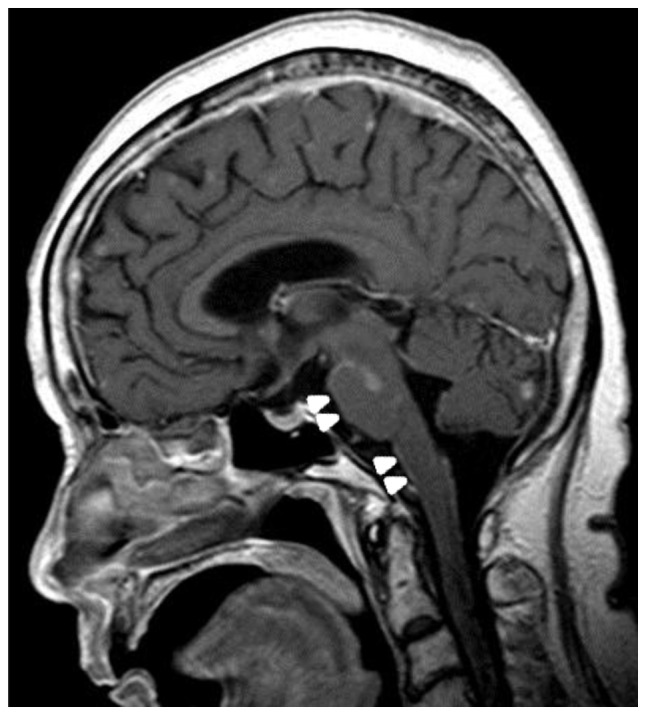
Gadlinium-enhanced T1WI magnetic resonance imaging on the 33rd day of hospitalization. The gadolinium-enhanced meninges in front of pons and medulla are shown (arrowheads).

**Figure 4:**
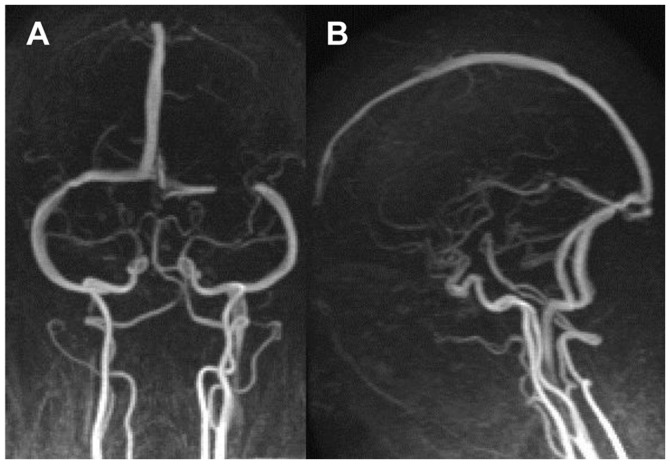
Magnetic resonance venography after four weeks of anticoagulation therapy. (A) Coronal and (B) saggital view. The cerebral venous sinuses are visible.

The diagnosis was later confirmed as a lung adenocarcinoma by an endobronchial biopsy, with a mutated (exon 19 deletion) epidermal growth factor receptor (EGFR). The patient also had multiple pulmonary, bone, and left adrenal metastases, and was therefore designated T4N3M1b, stage IV, by the clinical TNM classification system. There was temporary improvement of symptoms until the development of a severe headache on the 31st day of hospitalization, which became exacerbated with neck stiffness for a few days. On the 33rd day of hospitalization, brain CT revealed a hyperdense area at the right transverse venous sinus (Fig. 1). Magnetic resonance venography (MRV) revealed defects in the superior sagittal, right sigmoid, and right transverse venous sinuses, as well as the right internal jugular vein (Fig. 2). D-dimer levels were elevated from 0.2 to 1.3 μg/ml. The blood cell count, prothrombin time, activated partial thromboplastin time, fibrinogen, antithrombin III, protein C, protein S, and homocysteine levels were all within the normal limits, and lupus anticoagulant and anticardiolipin antibodies were negative. A neck echography did not detect lymph node metastases compressing the jugular vein. Anticoagulation therapy using unfractionated heparin was immediately administered following confirmation of the diagnosis of CVST. Brain MRI also demonstrated leptomeningeal gadolinium enhancement in the front of the pons and medulla (Fig. 3). The cerebrospinal fluid cell count, protein and CEA results increased to 12 cells/μl, 797 mg/dl and 4650 ng/ml, repsectively, whereas glucose results decreased to 11 mg/dl. Positive cerebrospinal fluid tumor cytology confirmed the diagnosis of LC. Following four weeks of anticoagulation therapy, complete thrombolysis was confirmed by MRV (Fig. 4). Anticoagulation therapy using warfarin was subsequently continued. The patient became drowsy due to secondary hydrocephalus; however, she returned to consciousness following insertion of a lumboperitoneal shunt. Effective treatment with gefitinib (250 mg daily) was administered, and the patient survived for 10 months following the diagnosis of CVST and LC.

## Discussion

CVST is a rare cerebrovascular disease with an estimated five people per million being affected each year, accounting for 0.5–1% of all cerebrovascular diseases. Causes of the disease are divided into acquired and genetic factors. Acquired factors include infection, surgery, trauma, pregnancy, puerperium, anti-phospholipid antibody syndrome, malignant tumor, and the use of hormone preparations. Genetic factors include a congenital clotting abnormality (3). Approximately 7.4% CVST cases are associated with malignant tumors. The underlying mechanisms of malignant tumor-induced CVST include direct pressure on the sinus venosus by the tumor (dural/cranial metastasis), direct tumor extension into the sinus venosus, thrombophilia, and side effects of anti-cancer agents such as tamoxifen and L-asparaginase (3). Sigsbee *et al* (4) reported seven malignant tumor patients with complications of superior sagittal venous sinus thrombosis. It was shown that thrombophilia due to the malignant tumor led to sinus thrombosis. Raizer *et al* (5) reported 20 malignant tumor patients with complications of CVST. According to their report, 9/20 patients had hematological malignancies, of whom five were administered L-asparaginase, two exhibited disseminated intravascular coagulation, and one had leukocytosis. In addition, 11/20 patients had solid tumors, six of which were dural/cranial metastases, three were administered antiestrogen agents, two had LC, one had low partial thromboplastin time, and one had thrombocytosis (5). The patient described in the present study exhibited a normal blood count at the time of onset and a slightly elevated D-dimer level of 1.3 ng/ml in the coagulation test. It was therefore unclear whether the patient had thrombophilia. Furthermore, there was no dural/cranial metastasis that would directly result in pressure on the sinus venosus.

Although the adverse effects of whole-brain radiotherapy are known, there have been no reports thus far on CVST complications caused by radiation exposure. At the time of hospitalization, the patient did not present with symptoms suggestive of LC, such as headache or neck stiffness, and there were no images to support a diagnosis of LC. The disease became evident during the progression of the patient, which was consistent with the onset of CVST. It is proposed that in this case, LC contributed to the onset of CVST. Previous case reports suggesting an association between LC and CVST include that of Li *et al* (6), who proposed that it was an extremely rare complication unique to patients with ovarian cancer. However, the underlying mechanism responsible for the disease was unclear and as with the present case study, pathology was not performed. Cerebrospinal fluid is produced in the choroid plexuses, circulated through the subarachnoid space, and absorbed in the sinus venosus through the arachnoid granulations protruding into the sinus venosus (7). Given this physiological circulatory route, it was inferred that thrombosis may occur due to direct extension into the sinus venosus of the tumor cells present in the cerebrospinal fluid, or the spread of inflammation into the vascular endothelium of the sinus venosus.

Approximately 90% of patients exhibit headache as a symptom of CVST, reflecting an increase in intracranial pressure. Pain throughout the whole head typically worsens over several days or weeks, although some patients develop sudden headaches suggestive of a subarachnoid hemorrhage, as was evident in the present case study. Neurological focal symptoms are not often present unless there are complications of cerebral infarction or cerebral hemorrhage (3). Abnormal findings indicating CVST using plain CT have a low detection rate of ~30%, therefore the current technique used for diagnosis is brain MRI (in particular a T2* MRI) combined with MRV (8). For treatment, anticoagulation therapy using unfractionated heparin or low-molecular-weight heparin is initially administered and thereafter is changed to vitamin K antagonists (3). According to a previous report by Ferro *et al* (9) on 624 patients, most of whom received anticoagulation therapy, 79% achieved complete neurologic recovery and 8.3% succummbed to the disease. Although cerebral infarction and cerebral hemorrhage may lead to poor prognosis, numerous patients receiving proper diagnosis and early treatment, can improve without experiencing any neurological sequelae.

Similar to hematological malignancies and breast cancer, lung cancer is often complicated by LC, with an incidence rate of 1–6%. LC is treated by a combination of systemic and intrathecal chemotherapy, and spinal cord radiation. The benefits of this treatment, however, are limited and even when administered, the prognosis is extremely poor with a median survival of 8–16 weeks (10). It has been reported that in EGFR mutation-positive (exon 19 deletion) patients with non-small cell lung cancer complicated by LC, a survival rate of 11 months can be achieved with targeted treatment using EGFR-tyrosine kinase inhibitors (TKI) such as gefitinib (11). Following the administration of gefitinib to the presented patient, a survival period from LC onset of ~10 months was achieved. Recent developments in the genetic diagnosis and targeted molecular therapy have significantly improved the prognosis of patients with non-small cell lung cancer. It has therefore become more viable to manage complications associated with lung cancer.

Based on a review of the literature, this is the first case report, to the best of our knowledge, of CVST concomitant with LC due to lung cancer. Adequate early diagnosis and treatment of CVST enabled an improved survival time, despite LC in this case. Development of headaches in patients with lung cancer should be considered for CVST. Furthermore, following diagnosis of CVST, LC should be investigated as an underlying cause.
